# Mitochondrial fission factor: a living way for mitochondria sensing food

**DOI:** 10.1002/mco2.770

**Published:** 2024-10-10

**Authors:** Mengmeng Zhi, Rong Cao, Xianghui Fu, Ling Li

**Affiliations:** ^1^ Department of Endocrinology Zhongda Hospital, School of Medicine, Southeast University Nanjing China; ^2^ Department of Biotherapy, Center for Diabetes and Metabolism Research, State Key Laboratory of Biotherapy and Cancer Center West China Hospital, Sichuan University and Collaborative Innovation Center Chengdu Sichuan China; ^3^ Department of Endocrinology, Institute of Glucose and Lipid Metabolism Southeast University Nanjing China

1

Recently, Henschke et al.^1^ revealed that protein kinase B/AKT (AKT)‐dependent phosphorylation of mitochondrial fission factor (MFF) in the liver triggered by pro‐opiomelanocortin (POMC) activation regulates mitochondrial fragmentation and glucose production, illustrating rapid signal transduction over the hypothalamus–liver axis in control of metabolic adaptation to anticipatory nutritional states.

A growing body of research expounds that anticipatory mechanisms in response to incoming nutrient are of essence to balance metabolic homeostasis, despite conventional homeostatic theories rely on internal feedback regulation. Predictive food cues are demonstrated to instantly trigger metabolic pathways in peripheral organs involving the liver and adipose tissue in order to increase nutrient availability. Thus, intensive probes into the principal mechanisms of specific peripheral adaptation to food‐sensing may bring profound influences on developing preventative strategies for metabolic diseases.

Notably, this latest study by Henschke et al.^1^ focused on the liver mitochondria, which is widely recognized as a nutrient sensor and regulatory hub for energy metabolism, and attempted to determine the roles and underlying mechanisms of liver mitochondrial dynamics in coordinating metabolic adaptation to predictive energy supply (Figure 1). Protein phosphorylation acts as a key sensor of nutrient metabolism to regulate intracellular signal transduction. The investigators first identified significantly up‐regulated phosphorylation level of serine 131 of MFF (MFFS131) in hepatic mitochondria exposed to caged food and refeeding via two unbiased phosphoproteomic screens. MFF has been engaged in boosted mitochondrial fission in a proved way of being phosphorylated at S155 and S172 depending on AMP‐activated protein kinase or protein kinase D, in response to energy stress and coupling mitochondrial fission to mitotic progression. Intriguingly, this study atypically defined AKT as the upstream trigger for MFF phosphorylation on a differential site (S131), unraveling a new pathway of AKT/MFFS131 that works during the cephalic phase. Attractively, blocking phosphorylation of MFFS131 impaired insulin sensitivity and dissipated insulin‐suppressed hepatic glucose production (HGP), suggesting a nonclassical AKT/MFFS131 pathway in control of HGP independent of the recognized glycogen synthase kinase‐3 beta. In line with this, recent work has revealed attenuated obesity‐associated mitochondrial fission and improved glucose metabolism in MFF‐deficient hepatocytes.^2^ These new findings shed light on potential targets for intervention in metabolic diseases such as diabetes and obesity.

**FIGURE 1:**
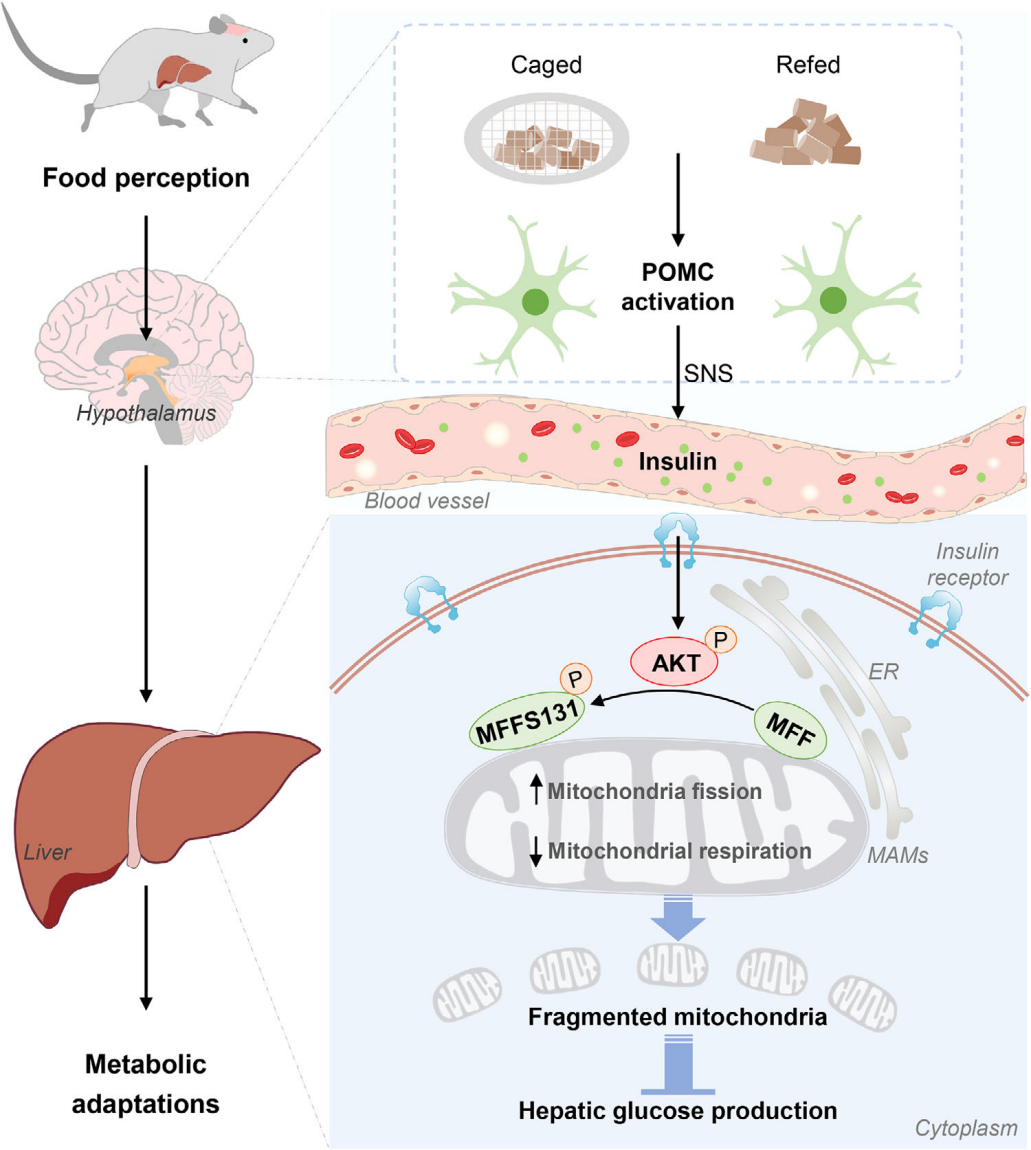
Hypothalamic POMC‐triggered hepatic MFFS131 phosphorylation links food perception to liver mitochondria fragmentation and metabolic adaptation. Both food sensation and food intake rapidly activate hypothalamic POMC‐expressing neurons, and then sympathetic nervous system (SNS) is excited to release norepinephrine, leading to elevated serum glucose and insulin. Subsequently, the insulin‐activated AKT localizes at mitochondria‐associated membranes (MAMs), phosphorylates MFFS131 on the outer membrane of mitochondria, and finally arouses mitochondrial fragmentation in the liver, which is essential for insulin‐stimulated suppression of HGP and metabolic adaptation to changing nutritional conditions.

Of note, rapid phosphorylation of MFFS131 and transient mitochondria fragments upon food perception can be also detected by hypothalamic POMC neuronal activation. It has been elaborated that instantaneous adaptive changes upon food sensation are driven by POMC neurons residing in arcuate nucleus. POMC‐driven neural pathways regulate food intake, energy expenditure, and systemic metabolism. Meanwhile, the alterations in the neural circuitry are causally linked to the development of metabolic diseases. The predominant perspective points that the activity of POMC is ruled by feedback control from the periphery in the light of nutritional state. In contrast, data in this study declared the transmission of feed‐forward signals from brain to periphery to balance imminent nutrition metabolism before food were consumed, which is likely attributed to POMC‐driven regulation of liver sympathetic and parasympathetic nerves. Supportively, Kwon et al.^3^ stated that POMC neurons innervated the liver via preganglionic parasympathetic to promote HGP. Furthermore, Brandt et al.^4^ claimed that POMC‐neuron activation upon sensory food perception promoted hepatic sympathetic innervation to induce endoplasmic reticulum remodeling in order to equilibrate energy homeostasis. Thereby, interventions on sympathetic or parasympathetic conduction pave a new path to improve glycemic control or insulin resistance in diabetes patients, raising the possibility of the emerging interventional operation endovascular denervation to treat diabetes and obesity.

It is important to identify the latent stimulator accelerating AKT activity. Herein, the observed activation of hepatic AKT signaling during the initial cephalic phase is probably regulated by a rapid and transient induction of circulating insulin in terms of the significantly coincident trends of change in phosphorylated AKT (p‐AKT) and insulin. Both of p‐AKT and serum insulin peaked at 5 min and then declined instantly during the period of food perception. Upon refeeding, the upregulation of p‐AKT and insulin triggered by stressed hyperglycemia continued going up, leading to further mitochondrial fragmentation. It is of interest in the future to clarify whether and how insulin signaling is truly involved in the process elicited by food perception and refeeding, particularly in the novel AKT/MFF pathway related to HGP.

Of concern, Henschke et al.^1^ further defined the dramatic morphological changes of mitochondria via performing transmission electron microscopy, and observed a flashy fragmentation of the mitochondrial network both in the early phase of sensing food and in the context of POMC stimulation. In addition, the inspired AKT‐dependent MFFS131 phosphorylation by food sensation was analogously proved to be essential for the hepatic mitochondria changes in both morphology and functionality. Mitochondria are highly dynamic organelles continuously undergoing fission and fusion to adapt to nutritional demands. Evidence suggests that during the development of metabolic abnormalities such as obesity and insulin resistance, mitochondria become more fragmented.^5^ The fragmented mitochondria lose the ability to supply energy for fat burning, further accelerating the process of fat deposition. Similarly, mitochondrial fragmentation in the liver upon sensing food mediated by insulin/AKT pathway blocked the energy generation for glucogenesis, and this AKT‐induced mitochondrial fragmentation depended on MFFS131 phosphorylation. A mutation that prevented phosphorylation of MFF abrogated AKT‐induced mitochondrial fragmentation, hinting a fresh signaling pathway regulating mitochondrial dynamics. For years, a mass of surveys focused on the role of mitochondrial repair in preventing and controlling metabolic diseases including diabetes, obesity and fatty liver disease. The latest investigation shows paradoxically reversed diet‐induced hepatosteatosis and obesity by inhibition of mammalian mitochondrial deoxyribonucleic acid. Besides, the novel antidiabetic agent named imeglimin was also shown to increase muscle glucose uptake and decrease HGP by targeting mitochondrial biogenesis. Hence, the accurate molecular connection between MFF‐dependent mitochondrial dynamics and the control of HGP indeed brings great potential to reveal new mechanisms and develop therapeutic strategies for obesity and type 2 diabetes mellitus.

In short, this study reveals a novel pathway through which sensory food anticipation orients peripheral organ to metabolic homeostasis. Looking forward, a bunch of energy‐related phosphoproteins in variety detected in the study and their underlying interactions deserve to be figured out. Broader excavations in the functional features of the phosphoproteome could offer holistic insights into the molecular signature of food‐sensing‐dependent control of peripheral function and glucolipid metabolism, and eventually carry profound therapeutic potentials in a wide range of metabolic disorders, especially in obesity and diabetes.

## AUTHOR CONTRIBUTIONS

X. F. and L. L. conceived the idea. M. Z. drafted the manuscript. R. C. designed the figure. L. L. and X. F. supervised and revised the manuscript. All authors have read and approved the final manuscript.

## CONFLICT OF INTEREST STATEMENT

None.

## ETHICS STATEMENT

Not applicable.

## Data Availability

Not applicable.

## References

[1] Henschke S , Nolte H , Magoley J , et al. Food perception promotes phosphorylation of MFFS131 and mitochondrial fragmentation in liver. Science. 2024;384(6694):438‐446. 38662831 10.1126/science.adk1005

[2] Hammerschmidt P , Ostkotte D , Nolte H , et al. CerS6‐derived sphingolipids interact with Mff and promote mitochondrial fragmentation in obesity. Cell. 2019;177(6):1536‐1552.e1523. 31150623 10.1016/j.cell.2019.05.008

[3] Kwon E , Joung HY , Liu SM , Chua SC Jr , Schwartz GJ , Jo YH . Optogenetic stimulation of the liver‐projecting melanocortinergic pathway promotes hepatic glucose production. Nat Commun. 2020;11(1):6295. 33293550 10.1038/s41467-020-20160-w PMC7722761

[4] Brandt C , Nolte H , Henschke S , et al. Food perception primes hepatic ER homeostasis via melanocortin‐dependent control of mTOR activation. Cell. 2018;175(5):1321‐1335.e1320. 30445039 10.1016/j.cell.2018.10.015 PMC6541012

[5] Kraus F , Roy K , Pucadyil TJ , Ryan MT . Function and regulation of the divisome for mitochondrial fission. Nature. 2021;590(7844):57‐66. 33536648 10.1038/s41586-021-03214-x

